# Prognostic Value of the C-Reactive Protein/Albumin Ratio and Systemic Immune-Inflammation Index for Patients With Colorectal Liver Metastasis Undergoing Curative Resection

**DOI:** 10.3389/pore.2021.633480

**Published:** 2021-03-24

**Authors:** Yuxiang Deng, Yujie Zhao, Jiayi Qin, Xiaozhen Huang, Ruomei Wu, Caixia Zhou, Zhizhong Pan

**Affiliations:** ^1^ Department of Colorectal Surgery, State Key Laboratory of Oncology in South China, Collaborative Innovation Center for Cancer Medicine, Sun Yat-sen University Cancer Center, Guangzhou, China; ^2^ Department of Radiation Oncology, Peking University Shenzhen Hospital, Shenzhen, China

**Keywords:** colorectal neoplasms, liver metastases, prognosis, CAR, SII

## Abstract

**Background:** We evaluated the prognostic value of C-reactive protein/albumin (CAR) and systemic immune-inflammation index (SII), which we calculated as neutrophil × platelet/lymphocyte) in patients with colorectal liver metastasis (CRLM) after curative resection.

**Methods:** We retrospectively enrolled 283 consecutive patients with CRLM who underwent curative resection between 2006 and 2016. We determined the optimal cutoff values of CAR and SII using receiver operating curve (ROC) analysis. Overall survival (OS)- and recurrence-free survival (RFS)-related to CAR and SII were analyzed using the log-rank test and multivariate Cox regression methods.

**Results:** We found that a high CAR was significantly associated with poor OS (P < 0.001) and RFS (P = 0.008) rates compared with a low CAR; a high SII was significantly associated with poor RFS (P = 0.003) rates compared with a low SII. The multivariate analysis indicated that CAR was an independent predictor of OS (hazard ratio [HR] = 2.220; 95% confidence interval [CI] = 1.387–3.550; P = 0.001) and RFS (HR = 1.494; 95% CI = 1.086–2.056; P = 0.014). The SII was an independent predictor of RFS (HR = 1.973; 95% CI = 1.230–3.162; P = 0.005) in patients with CRLM.

**Conclusion:** We proved that CAR was an independent predictor of OS and RFS in patients with CRLM who underwent curative resection, and that the prognostic value of CAR was superior to that of SII.

## Introduction

Colorectal cancer is one of the most common malignant tumors worldwide; its incidence and mortality are ranked 4th and 2nd, respectively [1]. The liver is the most frequent metastatic site for colorectal cancer, and 20–25% of patients have liver metastases at the time of their first diagnosis [2]. Although the treatment of patients with colorectal liver metastasis (CRLM) has improved in recent years, curative resection of liver metastases is still one of the most effective treatments [3]. However, approximately 75% of patients experience recurrence after the first liver metastasis resection, and only 10% of patients remain tumor-free within 10 years after liver metastasis resection [4, 5]. Due to the recurrence of tumors, most patients who have undergone liver metastasis resection are still unable to achieve long-term survival [6]. Therefore, there is an urgent need to identify effective prognostic factors to screen out high-risk patients and make clinical interventions to achieve optimal therapeutic outcomes.

Previous studies have shown that nutritional status and the inflammatory response play a key role in the development of cancer [7, 8]. Recent studies have indicated that CAR, the ratio of C-reactive protein to albumin, has a prognostic role in gastric cancer [9]. CAR can also predict the prognosis of patients with colorectal cancer [10]. Some meta-analyses have revealed that a high pretreatment CAR is an independent predictor of poor overall survival (OS) and disease-free survival/recurrence-free survival (DFS/RFS) in colorectal cancer patients [11–13]. The SII, the ratio of the product of neutrophils and platelets to lymphocytes, is associated with survival time in patients with esophageal cancer [14]. However, no research has explored the predictive value of CAR and SII in CRLM patients. We thus designed this study to examine the predictive value of CAR and SII in CRLM patients undergoing radical resection of primary tumors and liver metastases.

## Materials and Methods

We retrospectively analyzed patients with liver metastases of colorectal cancer at Sun Yat-sen University Cancer Center from March 2006 to December 2016. The enrollment criteria were as follows: 1) both primary and liver metastases underwent radical resection; 2) pathological biopsy-confirmed adenocarcinoma; and 3) preoperative metastases confined to the liver. The exclusion criteria were as follows: 1) missing routine blood or routine blood biochemical data before liver metastasis resection; 2) missing pathological or TNM staging data; and 3) incomplete follow-up within 3 months after surgery. Based on the above conditions, we ultimately enrolled 283 patients in our study. We classified the tumor stage according to the 2010 American Joint Committee on Cancer (AJCC) staging system. We conducted the study based on the ethical standards of the World Medical Association’s Declaration of Helsinki. The Ethics Committee of Sun Yat-sen University Cancer Center waived ethical approval due to the retrospective nature of the study; we kept all patient data private and documented the data confidentially.

Venous blood samples collected from the patients in the morning 7 days before surgery were used as conventional biochemical samples, analyzed by a Hitachi 7600 automatic biochemical analyzer. CAR is defined as the ratio of C-reactive protein to albumin in peripheral blood. Routine blood tests were performed using the Sysmex XE-5000TM automated blood system. SII is defined as the ratio of the product of neutrophils and platelets in peripheral blood to lymphocytes. Carcinoembryonic antigen (CEA) and carbohydrate antigen (CA19–9) were examined using an electroluminescence immunoassay analyzer.

Primary tumors were staged according to the seventh edition of the colorectal cancer UICC-TNM staging system. Colonoscopies, CT, magnetic resonance images (MRIs), and ultrasounds were used to assess the tumor condition before surgery. Treatment strategies for patients were developed by the multidisciplinary consultation team (MDT), including perioperative chemotherapy, radiofrequency ablation, and surgical treatment. If the patient’s tumor was considered resectable and the risk of recurrence was low, surgical treatment was directly recommended. All patients underwent standard complete mesocolic excision (CME) or total mesorectal excision (TME) and regional lymphadenectomy based on the tumor’s location. Perioperative chemotherapy regimens include CapeOX (oxaliplatin + capecitabine), FOLFOX (oxaliplatin + 5-fluorouracil + tetrahydrofolate), and FOLFIRI (irinotecan + 5-fluorouracil). The later lines of chemotherapy for most people were missing or unavailable from the follow-up data.

Patients were followed up every 3 months for the first 2 years after surgery, every 6 months for the third to fifth years, and annually thereafter. Evaluations at each visit included a physical examination, routine blood tests, and CEA and CA199 evaluations. Chest X-rays, abdominal and pelvic CT, colonoscopies, and pelvic MRIs were performed annually. OS was defined as the date of liver metastasis resection to the date of death due to any cause or the date of last follow-up. RFS was defined as the date of liver metastasis resection to recurrence, death, or last follow-up. Patients without any event (recurrence or death) at the last follow-up date were regarded as randomly censored. Follow-up was terminated in September 30, 2018.

We performed data processing and analysis using SPSS 20.0 software and GraphPad Prism 7 software. Receiver operating curve (ROC) analysis was applied to calculate the area under the ROC curve (AUC), which we used to determine the optimal cutoff value of CAR and SII. We drew the curve with the true positive rate (sensitivity) as the ordinate, the false positive rate (1-specificity) as the abscissa, and the Jordan index (sensitivity + specificity -1). The maximum value was defined as the optimal cutoff value. The Kaplan-Meier and log-rank methods were employed to scrutinize postoperative survival. Comparison between groups was performed using the χ^2^ test or Fisher’s test. To avoid collinearity, if the P values of the variables and CAR or SII in the χ^2^ test were more than 0.05, and the P values in the Cox regression model for univariate analysis were less than 0.05, then the variable would be included in the multivariate analysis. Hazard ratios (HRs) and 95% confidence intervals (CI) were calculated. P < 0.05 was considered to be statistically significant.

## Results

### Optimal Cutoff Values of Variables

Using the ROC curves for OS and RFS, the optimal cutoff values for age, the diameter and the number of the liver metastases were both 69 years old, 3 cm, and 1. We dichotomized the patients into two groups using the above cutoff values. The cutoff values of CAR for OS and RFS were 0.0322 and 0.0369 by ROC analysis, separately. When analyzing the association of CAR and OS, the patients were divided into a low CAR group (CAR ≤ 0.0322), and a high CAR group (CAR > 0.0322) according to the optimal cutoff values. When analyzing CAR and RFS, patients were divided into a low CAR group (CAR ≤ 0.0369), and a high CAR group (CAR > 0.0369). The cutoff values of SII for OS and RFS were both 0.0135.

### The Relationship Between C-Reactive Protein/Albumin or Systemic Immune-Inflammation Index and Clinicopathological Characteristics

Among the 283 patients enrolled, the mean CAR and SII values were 0.2290 and 0.0303, and the standard deviations were 0.6401 and 0.0302, respectively. There were 96 (33.9%) females and 187 (66.1%) males; the median age was 57 years (range: 25–82 years). Regarding the T stage of the primary tumor, there was 1 case of T1, 26 of T2, 156 of T3, and 100 of T4. For the N stage, there were 120 cases of N0, 105 of N1, and 58 of N2. The median diameter of the largest liver metastases was 2.4 cm (range: 0.3–11.0 cm). The median values of C-reactive protein, albumin, neutrophils, platelets and lymphocytes were 2.23 mg/L (range: 0.13–225.77 mg/L), 41.90 g/L (range: 12.20–77.05 g/L), 3.2 ✕ 10^9^/L (range: 0.8–24.6 ✕ 10^9^/L), 204.0 ✕ 10^9^/L (range: 31.3–750.0 ✕ 10^9^/L) and 1.6 ✕ 10^9^/L (range: 0.1–4.2 ✕ 10^9^/L), respectively.

As depicted in Table 1, When the cutoff value of CAR was 0.0322, patients older than 69 years old (*p* = 0.002), with primary lesions in the colon (*p* = 0.004), with liver metastasis tumors larger than 3 cm (*p* = 0.002) and those who received synchronous hepatic resection (*p* = 0.015) were more likely to belong to the high CAR group. When the cutoff value of CAR was 0.0369, patients older than 69 years old (*p* = 0.007), with primary lesions in the colon (*p* = 0.025), higher N stage (*p* = 0.048), with liver metastasis tumors larger than 3 cm (*p* < 0.001), those who received synchronous hepatic resection (*p* = 0.019), higher pre-operative CA19–9 (*p* = 0.028) were more likely to belong to the high CAR group. As shown in Table 2, male patients (*p* = 0.021), with liver metastasis tumors larger than 3 cm (*p* = 0.045), and those with multiple liver metastases (*p* = 0.005) were more likely to belong to the high SII group. There was no significant association between CAR or SII and the T stage, pathological differentiation, pre- or post-operative chemotherapy, and CEA levels before liver metastasis resection, indicating no collinearity among the independent variables.

**TABLE 1:** Clinical characteristics of the patients stratified by CAR.

Characteristics	n (%)	CAR					
		≤0.0322	>0.0322	*p*	≤0.0369	>0.0369	*p*
**Age (years)**							
≤69	251 (88.7)	91 (96.8)	160 (84.7)	0.002	101 (95.3)	150 (84.7)	0.007
>69	32 (11.3)	3 (3.2)	29 (15.3)		5 (4.7)	27 (15.3)	
**Sex**							
Female	96 (33.9)	35 (37.2)	61 (32.3)	0.407	39 (36.8)	57 (32.2)	0.430
Male	187 (66.1)	59 (62.8)	128 (67.7)		67 (63.2)	120 (67.8)	
**Primary tumor site**							
Colon	186 (65.7)	51 (54.3)	135 (71.4)	0.004	61 (57.5)	125 (70.6)	0.025
Rectum	97 (34.3)	43 (45.7)	54 (28.6)		45 (42.5)	52 (29.4)	
**T Stage**							
1–3	183 (64.7)	59 (62.8)	124 (65.6)	0.638	67 (63.2)	116 (65.5)	0.692
4	100 (35.3)	35 (37.2)	65 (34.4)		39 (36.8)	61 (34.5)	
**N stage**							
0	120 (42.4)	35 (37.2)	85 (45.0)	0.215	37 (34.9)	83 (46.9)	0.048
1–2	163 (57.6)	59 (62.8)	104 (55.0)		69 (65.1)	94 (53.1)	
**Histological grade**							
Well/moderate	251 (88.7)	85 (90.4)	166 (87.8)	0.516	96 (90.6)	155 (87.6)	0.441
Poor	32 (11.3)	9 (9.6)	23 (12.2)		10 (9.4)	22 (12.4)	
**Liver metastases tumor size (cm)**							
≤3	197 (69.6)	77 (81.9)	120 (63.5)	0.002	88 (83.0)	109 (61.6)	<0.001
>3	86 (30.4)	17 (18.1)	69 (36.5)		18 (17.0)	68 (38.4)	
**Liver metastases number**							
Single	135 (47.7)	46 (48.9)	89 (47.1)	0.770	51 (48.1)	84 (47.5)	0.915
Multiple	148 (52.3)	48 (51.1)	100 (52.9)		55 (51.9)	93 (52.5)	
**Hepatic resection timing**							
Metachronous	119 (42.0)	49 (52.1)	70 (37.0)	0.015	54 (50.9)	65 (36.7)	0.019
Synchronous	164 (58.0)	45 (47.9)	119 (63.0)		52 (49.1)	112 (63.3)	
**Preoperative chemotherapy**							
Yes	143 (50.5)	46 (48.9)	97 (51.3)	0.705	53 (50.0)	90 (50.8)	0.890
No	140 (49.5)	48 (51.1)	92 (48.7)		53 (50.0)	87 (49.2)	
**Postoperative chemotherapy**							
Yes	218 (77.0)	78 (83.0)	140 (74.1)	0.093	87 (82.1)	131 (74.0)	0.119
No	65 (23.0)	16 (17.0)	49 (25.9)		19 (17.9)	46 (26.0)	
**CEA (ng/ml)**							
≤5	118 (41.7)	44 (46.8)	74 (39.2)	0.219	50 (47.2)	68 (38.4)	0.148
>5	165 (58.3)	50 (53.2)	115 (60.8)		56 (52.8)	109 (61.6)	
**CA199 (U/mL)**							
≤37	213 (75.3)	75 (79.8)	138 (73.0)	0.161	87 (82.1)	126 (71.2)	0.028
>37	69 (24.4)	18 (19.1)	51 (27.0)		18 (17.0)	51 (28.8)	
Not available	1 (0.4)	1 (1.1)	0 (0)		1 (0.9)	0 (0)	
**C-reactive protein (mg/L)**							
≤3	166 (58.7)	94 (100.0)	72 (38.1)	<0.001	106 (100.0)	60 (33.9)	<0.001
>3	117 (41.3)	0 (0)	117 (61.9)		0 (0)	117 (66.4)	
**Albumin (g/L)**							
<40	75 (26.5)	9 (9.6)	66 (34.9)	<0.001	15 (14.2)	60 (33.9)	<0.001
40–55	207 (73.1)	84 (89.4)	123 (65.1)		90 (84.9)	117 (66.1)	
>55	1 (0.4)	1 (1.1)	0 (0)		1 (0.9)	0 (0)	
**Neutrophil (✕10** ^**9**^ **/L)**							
<1.8	23 (8.1)	10 (10.6)	13 (6.9)	0.059	12 (11.3)	11 (6.2)	0.017
1.8–6.3	246 (86.9)	83 (88.3)	163 (86.2)		93 (87.7)	153 (86.4)	
>6.3	14 (4.9)	1 (1.1)	13 (6.9)		1 (0.9)	13 (7.3)	
**Platelet (✕10** ^**9**^ **/L)**							
<100	4 (1.4)	2 (2.1)	2 (1.1)	0.278	2 (1.9)	2 (1.1)	0.451
100–300	240 (84.8)	83 (88.3)	157 (83.1)		93 (87.7)	157 (83.1)	
>300	39 (13.8)	9 (9.6)	30 (15.9)		11 (10.4)	28 (15.9)	
**Lymphocyte (✕10** ^**9**^ **/L)**							
<1.1	39 (13.8)	13 (13.8)	26 (13.8)	0.381	13 (12.3)	26 (14.7)	0.211
1.1–3.2	239 (84.5)	81 (86.2)	158 (83.6)		93 (87.7)	146 (82.5)	
>3.2	5 (1.8)	0 (0)	5 (2.6)		0 (0)	5 (2.8)	
**SII**							
≤0.0135	45 (15.9)	19 (20.2)	26 (13.8)	0.162	20 (18.9)	25 (14.1)	0.291
>0.0135	238 (84.1)	75 (79.8)	163 (86.2)		86 (81.1)	152 (85.9)	

**Abbreviations:** CEA, carcinoembryonic antigen; CA19–9, cancer antigen 19–9.

**TABLE 2:** Clinical characteristics of the patients stratified by SII.

Characteristics	n (%)	SII		
		≤0.0135	>0.0135	*p*
**Age (years)**				
≤69	251 (88.7)	42 (93.3)	209 (87.8)	0.284
>69	32 (11.3)	3 (6.7)	29 (12.2)	
**Sex**				
Female	96 (33.9)	22 (48.9)	74 (31.1)	0.021
Male	187 (66.1)	23 (51.1)	164 (68.9)	
**Primary tumor site**				
Colon	186 (65.7)	30 (66.7)	156 (65.5)	0.885
Rectum	97 (34.3)	15 (33.3)	82 (34.5)	
**T Stage**				
1–3	183 (64.7)	29 (64.4)	154 (64.7)	0.973
4	100 (35.3)	16 (35.6)	84 (35.3)	
**N stage**				
0	120 (42.4)	19 (42.2)	101 (42.4)	0.979
1–2	163 (57.6)	26 (57.8)	137 (57.6)	
**Histological grade**				
Well/moderate	251 (88.7)	40 (88.9)	211 (88.7)	0.964
Poor	32 (11.3)	5 (11.1)	27 (11.3)	
**Liver metastases tumor size (cm)**				
≤3	197 (69.6)	37 (82.2)	160 (67.2)	0.045
>3	86 (30.4)	8 (17.8)	78 (32.8)	
**Liver metastases number**				
Single	135 (47.7)	30 (66.7)	105 (44.1)	0.005
Multiple	148 (52.3)	15 (33.3)	133 (55.9)	
**Hepatic resection timing**				
Metachronous	119 (42.0)	13 (28.9)	106 (44.5)	0.051
Synchronous	164 (58.0)	32 (71.1)	132 (55.5)	
**Preoperative chemotherapy**				
Yes	143 (50.5)	22 (48.9)	121 (50.8)	0.810
No	140 (49.5)	23 (51.1)	117 (49.2)	
**Postoperative chemotherapy**				
Yes	218 (77.0)	36 (80.0)	182 (76.5)	0.606
No	65 (23.0)	9 (20.0)	56 (23.5)	
**CEA (ng/ml)**				
≤5	118 (41.7)	24 (53.3)	94 (39.5)	0.084
>5	165 (58.3)	21 (46.7)	144 (60.5)	
**CA199 (U/mL)**				
≤37	213 (75.3)	35 (77.8)	178 (74.8)	0.702
>37	69 (24.4)	10 (22.2)	59 (24.8)	
Not available	1 (0.4)	0 (0)	1 (0.4)	
**C-reactive protein (mg/L)**				
≤3	166 (58.7)	29 (64.4)	137 (57.6)	0.390
>3	117 (41.3)	16 (35.6)	101 (42.4)	
**Albumin (g/L)**				
<40	75 (26.5)	18 (40.0)	57 (23.9)	0.077
40–55	207 (73.1)	27 (60.0)	180 (75.6)	
>55	1 (0.4)	0 (0)	1 (0.4)	
**Neutrophil (✕10** ^**9**^ **/L)**				
<1.8	23 (8.1)	14 (31.1)	9 (3.8)	<0.001
1.8–6.3	246 (86.9)	31 (68.9)	215 (90.3)	
>6.3	14 (4.9)	0 (0)	14 (5.9)	
**Platelet (✕10** ^**9**^ **/L)**				
<100	4 (1.4)	0 (0)	4 (1.7)	0.037
100–300	240 (84.8)	33 (73.3)	207 (87.0)	
>300	39 (13.8)	12 (26.7)	27 (11.3)	
**Lymphocyte (✕10** ^**9**^ **/L)**				
<1.1	39 (13.8)	21 (46.7)	18 (7.6)	<0.001
1.1–3.2	239 (84.5)	24 (53.3)	215 (90.3)	
>3.2	5 (1.8)	0 (0)	5 (2.1)	

**Abbreviations:** CEA, carcinoembryonic antigen; CA19-9, cancer antigen 19-9.

### Association Between C-Reactive Protein/Albumin or Systemic Immune-Inflammation Index and Survival

**FIGURE 1:**
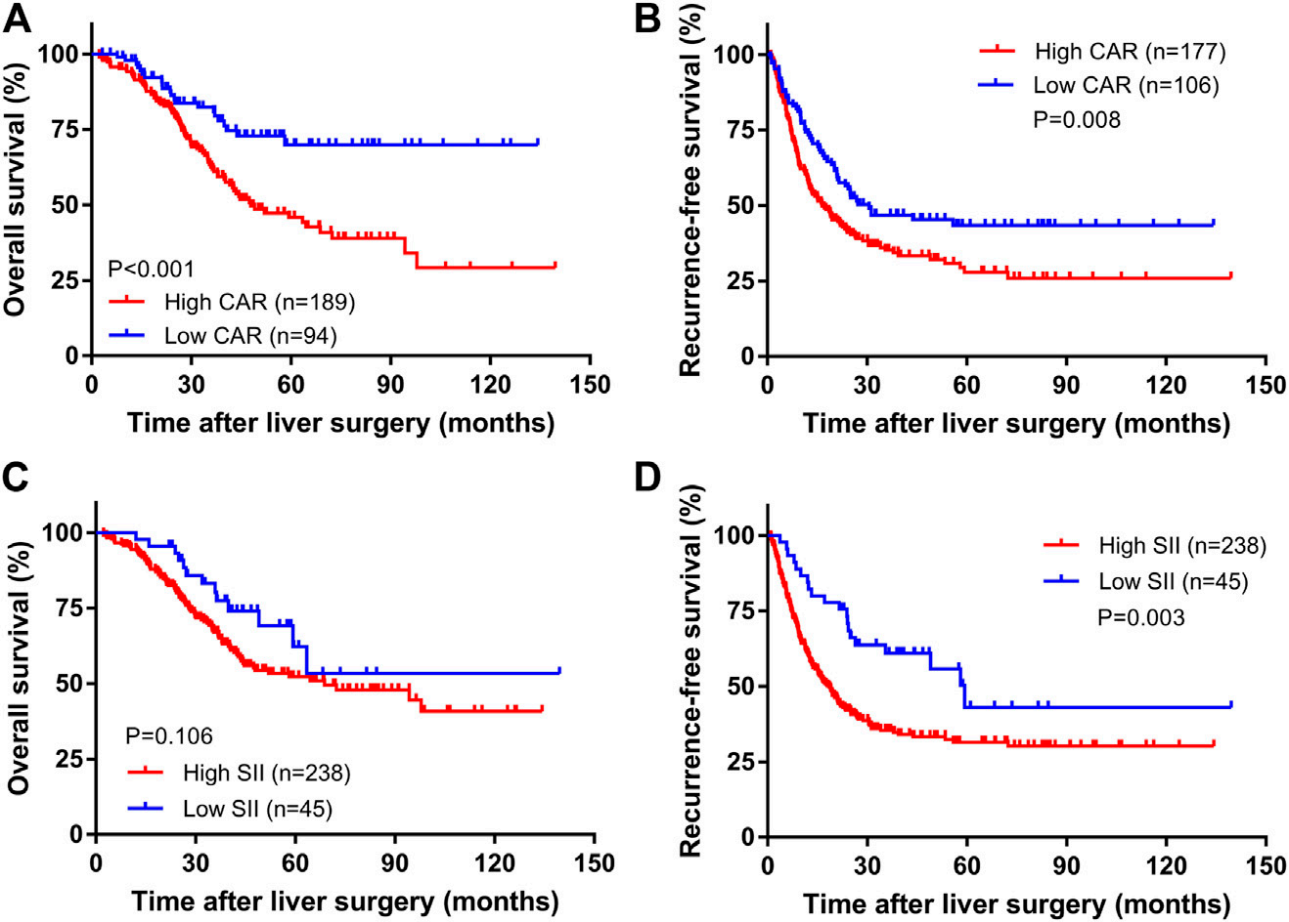
The Kaplan–Meier curves of CAR for OS **(A)** and RFS **(B)** and SII for OS **(C)** and RFS **(D)**.

The median follow-up time was 35.4 months (range: 2.3–139.6 months). A total of 107 patients died, and 139 relapsed during the follow-up period. The overall survival rates for all patients at 1, 3, and 5 years were 95.0%, 69.2%, and 54.0%, and the total RFS rates were 64.6%, 39.5%, and 33.8%, respectively. Kaplan-Meier survival analysis showed that the median OS times of the high CAR group and the low CAR group were 36.7 and 39.6 months, respectively; the median relapse-free survival times were 17.1 and 25.1 months, respectively. The 1-, 3-, and 5-years OS rates for the high and low CAR groups were 93.6% and 97.8%, 62.6% and 82.3%, 45.8% and 69.9%, respectively. The overall RFS rates at 1, 3, and 5 years were 58.8% and 74.4%, 35.2% and 46.7%, and 27.9% and 43.4%, respectively. The differences in the OS and RFS were statistically significant (*p* < 0.001, Figure 1A; *p* = 0.008, Figure 1B).

**FIGURE 2:**
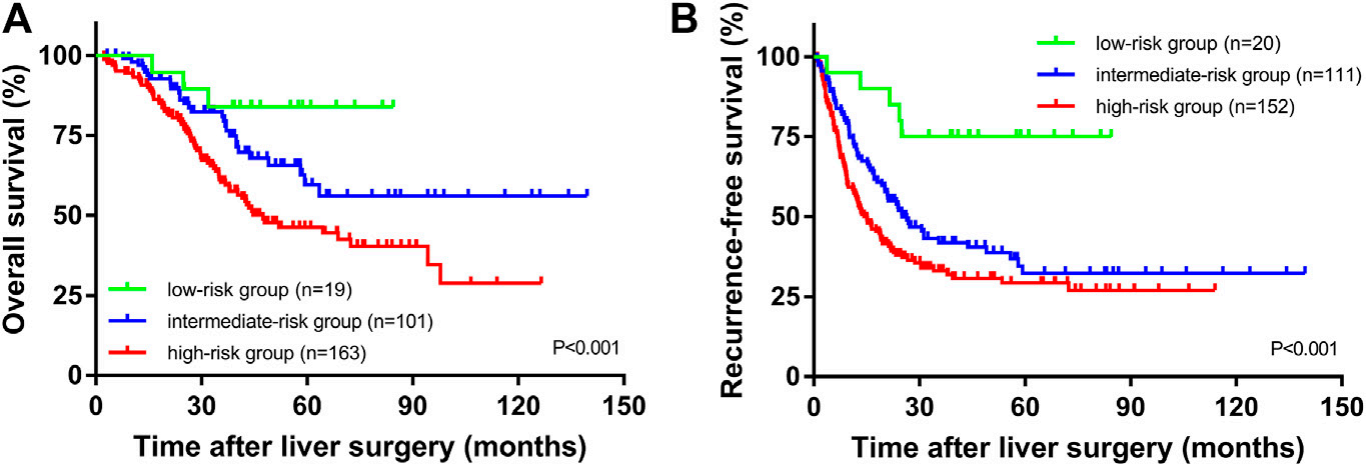
OS **(A)** and RFS **(B)** of CRLM patients according to the combination of CAR and SII.

The median OS times of the high SII group and the low SII group were 33.5 and 39.9 months, respectively. The median relapse-free survival times were 16.9 and 35.4 months, respectively. The difference in OS time between the high and low SII groups was not statistically significant (*p* = 0.106, Figure 1C). The RFS rates for the high and low SII groups at 1, 3, and 5 years were 60.9% and 84.4%, 35.4% and 60.9%, and 31.5% and 43.1%, respectively; the difference was statistically significant (*p* = 0.003, Figure 1D). In addition, we derived a risk scoring system named the combined marker based on CAR and SII factors according to the following criteria: low-risk group = 0 factor presence a higher level; intermediate-risk group = 1 factor presence a higher level; and high-risk group = 2 factors both presence higher levels. There were significant differences in OS (*p* < 0.001; Figure 2A) and RFS (*p* < 0.001; Figure 2B) among the three groups stratified by risk scoring.

### Univariate and Multivariate Analyses of Prognostic Factors

As displayed in Table 3, univariate Cox regression analysis revealed that the pre-operative chemotherapy (*p* < 0.001), and preoperative CAR (*p* = 0.001) were all associated with patient OS. The pre-operative chemotherapy (*p* < 0.001), CEA levels before surgery (*p* = 0.004), CAR (*p* = 0.009) and SII (*p* = 0.003) were associated with RFS.

**TABLE 3:** Univariate and multivariate analyses using Cox proportional hazards models using factors influencing OS and RFS.

	OS				RFS			
	Univariate analysis		Multivariate analysis		Univariate analysis		Multivariate analysis	
	HR (95%CI)	P Value	HR (95%CI)	P Value	HR (95%CI)	P Value	HR (95%CI)	P Value
**Age (year)**								
>69 vs. ≤69	2.902 (1.826–4.610)	<0.001			1.396 (0.906–2.148)	0.130		
**Gender**								
Male vs. Female	1.253 (0.830–1.889)	0.283			1.088 (0.794–1.491)	0.600		
**Tumor site**								
Rectum vs. Colon	1.729 (1.180–2.533)	0.005			1.242 (0.915–1.685)	0.164		
**T Stage**								
4 vs. 1–3	1.380 (0.939–2.029)	0.102			1.255 (0.925–1.702)	0.145		
**N stage**								
1–2 vs.0	1.855 (1.234–2.789)	0.003			1.867 (1.364–2.555)	<0.001		
**Histological grade**								
Poor vs. Well/moderate	1.419 (0.834–2.416)	0.197			1.192 (0.755–1.882)	0.452		
**Liver metastases tumor size (cm)**								
>3 vs. ≤3	1.918 (1.306–2.818)	0.001			1.757 (1.290–2.394)	<0.001		
**Liver metastases number**								
Multiple vs. Single	1.824 (1.232–2.702)	0.003			2.006 (1.477–2.725)	<0.001		
**Hepatic resection timing**								
Synchronous vs. Metachronous	0.798 (0.546–1.166)	0.244			0.736 (0.547–0.991)	0.043		
**Preoperative chemotherapy**								
Yes vs. No	2.174 (1.463–3.228)	<0.001	2.132 (1.435–3.166)	<0.001	2.489 (1.827–3.390)	<0.001	2.605 (1.909–3.553)	<0.001
**Postoperative chemotherapy**								
Yes vs. No	0.677 (0.431–1.064)	0.091			1.202 (0.830–1.742)	0.331		
**CEA (ng/ml)**								
>**5 vs.** ≤**5**	1.427 (0.950–2.144)	0.087			1.607 (1.167–2.213)	0.004	1.410 (1.031–1.928)	0.031
**CA199 (U/mL)**								
>37 vs. ≤37	1.095 (0.683–1.757)	0.706			1.250 (0.856–1.824)	0.248		
**CAR**								
>0.0322 vs. ≤0.0322	2.270 (1.419–3.632)	0.001	2.220 (1.387–3.550)	0.001	1.528 (1.112–2.101)	0.009	1.494 (1.086–2.056)	0.014
**SII**								
>0.0135 vs. ≤0.0135	1.607 (0.899–2.872)	0.109			2.023 (1.269–3.226)	0.003	1.973 (1.230–3.162)	0.005

**Abbreviations:** HR, hazard ratio; CI, confidence interval; CEA, carcinoembryonic antigen; CA19–9, cancer antigen 19–9.

Note: Comparison between groups was performed using the χ^2^ test or Fisher’s test. To avoid collinearity, if the P values of the variables and CAR or SII in the χ^2^ test were more than 0.05, and the P values in the Cox regression model for univariate analysis were less than 0.05, then the variable would be included in the multivariate analysis.

Multivariate Cox regression analysis indicated that receiving chemotherapy before hepatic resection (*p* < 0.001), and a higher pre-operative CAR (*p* = 0.001) were all independent risk factors affecting OS. Receiving chemotherapy before hepatic resection (*p* < 0.001), preoperative higher CEA levels (*p* = 0.031), CAR (*p* = 0.014), and SII (*p* = 0.005) were independent risk factors affecting RFS.

Table 4 explored whether the combined marker was an independent prognostic factor of OS and RFS. Because the P values of χ^2^ test results of the age, primary tumor site, and the size of the liver metastases with the combined marker were less than 0.05, they were not included in uni- and multivariate COX analysis to avoid collinearity (Supplementary Table S1).

**TABLE 4:** Univariate and multivariate analyses using Cox proportional hazards models using factors influencing OS and RFS.

	OS				RFS			
	Univariate analysis		Multivariate analysis		Univariate analysis		Multivariate analysis	
	HR (95%CI)	P Value	HR (95%CI)	P Value	HR (95%CI)	P Value	HR (95%CI)	P Value
**Age (year)**								
>69 vs. ≤69	2.902 (1.826–4.610)	<0.001			1.396 (0.906–2.148)	0.130		
**Gender**								
Male vs. Female	1.253 (0.830–1.889)	0.283			1.088 (0.794–1.491)	0.600		
**Tumor site**								
Rectum vs. Colon	1.729 (1.180–2.533)	0.005			1.242 (0.915–1.685)	0.164		
**T Stage**								
4 vs. 1–3	1.380 (0.939–2.029)	0.102			1.255 (0.925–1.702)	0.145		
**N stage**								
1–2 vs.0	1.855 (1.234–2.789)	0.003	1.968 (1.306–2.964)	0.001	1.867 (1.364–2.555)	<0.001	2.187 (1.588–3.013)	<0.001
**Histological grade**								
Poor vs. Well/moderate	1.419 (0.834–2.416)	0.197			1.192 (0.755–1.882)	0.452		
**Liver metastases tumor size (cm)**								
>3 vs. ≤3	1.918 (1.306–2.818)	0.001			1.757 (1.290–2.394)	<0.001		
**Liver metastases number**								
Multiple vs. Single	1.824 (1.232–2.702)	0.003	-	0.216	2.006 (1.477–2.725)	<0.001	1.429 (1.029–1.984)	0.033
**Hepatic resection timing**								
Synchronous vs. Metachronous	0.798 (0.546–1.166)	0.244			0.736 (0.547–0.991)	0.043	-	0.901
**Preoperative chemotherapy**								
Yes vs. No	2.174 (1.463–3.228)	<0.001	2.205 (1.484–3.276)	<0.001	2.489 (1.827–3.390)	<0.001	2.465 (1.765–3.442)	<0.001
**Postoperative chemotherapy**								
Yes vs. No	0.677 (0.431–1.064)	0.091			1.202 (0.830–1.742)	0.331		
**CEA (ng/ml)**								
>**5 vs.** ≤**5**	1.427 (0.950–2.144)	0.087			1.607 (1.167–2.213)	0.004	1.434 (1.048–1.961)	0.024
**CA199 (U/mL)**								
>37 vs. ≤37	1.095 (0.683–1.757)	0.706			1.250 (0.856–1.824)	0.248		
**CAR + SII**								
low-Risk	1 (References)	0.003	1 (References)	0.002	1 (References)	0.001	1 (References)	<0.001
intermediate-Risk	2.302 (0.701–7.566)		2.260 (0.687–7.433)		3.228 (1.300–8.018)		3.440 (1.381–8.572)	
high-Risk	4.141 (1.304–13.144)		4.245 (1.337–13.481)		4.485 (1.826–11.016)		5.212 (2.106–12.900)	

**Abbreviations:** HR, hazard ratio; CI, confidence interval; CEA, carcinoembryonic antigen; CA19-9, cancer antigen 19-9.

Note: Comparison between groups was performed using the χ^2^ test or Fisher’s test. To avoid collinearity, if the P values of the variables and CAR or SII in the χ^2^ test were more than 0.05, and the P values in the Cox regression model for univariate analysis were less than 0.05, then the variable would be included in the multivariate analysis.

Multivariate Cox regression analysis indicated that a higher N stage (*p* = 0.001 and *p* < 0.001), receiving chemotherapy before hepatic resection (*p* < 0.001 and *p* < 0.001), and patient in high-risk group (*p* = 0.002 and p < 0.001) were all independent risk factors affecting OS and RFS. Besides, multiple liver metastases (*p* = 0.033) and preoperative higher CEA levels (*p* = 0.024) were also independent risk factors for RFS.

## Discussion

Approximately 150 years ago, researchers found a large amount of leukocyte infiltration in tumor tissues, which means that cancer has a certain correlation with chronic inflammation [15]. Studies have demonstrated that inflammation plays an important role in the pathogenesis, development, and therapeutic response of many tumors, and can contribute to tumor proliferation, angiogenesis, and metastasis by affecting the body’s immune response [7, 16, 17]. C-reactive protein is a biomarker of the inflammatory response that is produced by the liver; at increased levels, it may cause the body to create a microenvironment that is conducive to tumor cell proliferation and metastasis [18]. Thomsen et al. found that higher C-reactive protein levels were also an unfavorable prognostic factor in colorectal cancer patients; this result was confirmed by the meta-analysis of Woo et al. [19, 20]. The above research suggests that C-reactive protein is not only a common inflammatory marker, but also an effective prognostic indicator for malignant tumors. At the same time, the nutritional status of cancer patients will have a greater impact on their prognosis. Serum albumin is a protein produced by the liver, and is often used clinically as a benchmark of patients’ nutritional status; its level can also reflect the severity of disease. This marker is inversely related to post-operative mortality in patients with bowel cancer [21]. Studies have shown that lower albumin levels before surgery increase the overall mortality and risk of cancer-specific deaths [22]. In addition, higher levels of albumin can improve the body’s immune system, including cellular and humoral immunity [23]. Based on the above information, we can consider both elevated C-reactive protein and reduced albumin as risk factors in tumor development and combine these two indicators; namely, the CAR value. The CAR value can reflect patients’ immune function and nutritional status. Studies have shown that CAR could be a predictive marker for metastatic colorectal cancer patients. Shibutani et al. found CAR was a useful indicator for predicting the chemotherapeutic outcome in patients with metastatic CRC treated with later-line chemotherapy [24]. Sakamoto et al. indicated that OS and RFS were significantly better in low-CAR groups than in high-CAR groups [25]. According to our results, a high CAR is an unfavorable prognostic factor for patients with liver metastases from colorectal cancer. This outcome is consistent with previous researches. Moreover, we also found a predictive value of SII in our study. We identified a subgroup of patients with both higher CAR and SII that had the worst OS and RFS rates, implying that a combination of the scores of these two markers may be a better survival indicator in CRLM patients undergoing curative resection.

We explored the predictive value of the SII—a combination of neutrophil, lymphocyte, and platelet levels—which has a stronger predictive value than the neutrophil to lymphocyte ratio (NLR) and monocyte to lymphocyte ratio (PLR) in hepatocellular carcinoma [26]. In our study, the SII level was a risk factor for RFS in CRLM patients. This may have been due to a rise in neutrophil and platelet levels, leading to an increase in vascular endothelial growth factor (VEGF), which promotes the generation of blood vessels and tumor progression [27]. In addition, lymphocytes are a vital component of the body’s immune response function, which affects tumor progression by secreting cytokines and inducing cell death [28]. A decline in the number of lymphocyte levels means that the patient lacks an immune reaction, which culminates in tumor progression. Therefore, when the SII level is higher, this may signal a poor prognosis. However, we also found that the SII is not a risk factor for overall patient survival, which may be because this value is not important enough for OS. This suggests that CAR is more valuable for predicting the prognosis of bowel cancer liver metastasis. Zhou et al. evaluated the prognostic potential of the post-operative scores of inflammation indexes (including CAR and SII) in colorectal cancer patients. They discovered that CAR and SII were associated with poor OS and PFS [29]. Nevertheless, they focused on the levels of post-operative SII and CAR, rather than the pre-operative level in our study. Our meaningful results showed that patients can be stratified before surgery to guide subsequent treatment. Moreover, we identified a subgroup of patients with both higher CAR and SII that had the worst OS and RFS rates, implying that a combination of the scores of these two markers may be a better survival indicator in CRLM patients undergoing curative resection.

For patients with CRLM, CT, MRI, and genetic testing can be used to monitor disease progression and to predict patient outcomes. That said, these detection methods are both expensive and inconvenient. CAR and SII are both low-cost, non-invasive indicators that can not only be detected repeatedly, but are also easy to perform. As prognostic predictors for CRLM patients, CAR and SII can help clinicians to distinguish between high- and low-risk patients, and to design appropriate treatment strategies to obtain the optimal therapeutic effect before surgery.

Our study has some limitations. First, we collected data from a single institution, so our conclusions may be biased. Second, for different investigations, the cutoff values of CAR and SII are different. We carried out a retrospective study. As such, it will be necessary to determine the optimal cutoff value in future prospective research with a larger sample size.

## Conclusion

In sum, our findings indicated that CAR is an independent prognostic indicator of OS and RFS in patients with CRLM after curative resection, and that its prognostic value is better than that of SII.

## Data Availability

The datasets presented in this study can be found in online repositories. The names of the repository/repositories and accession number(s) can be found below: The authenticity of this article has been validated by uploading the key raw data onto the Research Data Deposit public platform (http://www.researchdata.org.cn), with the approval number as RDDA2020001421.
