# Supplementary material for: Prognostic Value of the C-Reactive Protein/Albumin Ratio and Systemic Immune-Inflammation Index for Patients With Colorectal Liver Metastasis Undergoing Curative Resection
Source: Pathol Oncol Res. 2021 Mar 24;27:633480. doi: 10.3389/pore.2021.633480 (PMC8262228; doi:10.3389/pore.2021.633480)
Supplement: Supplementary file 1 [file Table1.DOCX]

**Supplementary Table 1.** Clinical characteristics of the patients stratified by CAR+SII

| Characteristics | n (%) | CAR+SII | | | |
| --- | --- | --- | --- | --- | --- |
|  |  | low-risk | intermediate-risk | high-risk | P |
| **Age (years)** |  |  |  |  |  |
| ≤69 | 251 (88.7) | 19 (100) | 95 (94.1) | 137 (84.0) | 0.012 |
| >69 | 32 (11.3) | 0 (0) | 6 (5.9) | 26 (16.0) |  |
| **Sex** |  |  |  |  |  |
| Female | 96 (33.9) | 11 (57.9) | 35 (34.7) | 50 (30.7) | 0.059 |
| Male | 187 (66.1) | 8 (42.1) | 66 (65.3) | 113 (69.3) |  |
| **Primary tumor site** |  |  |  |  |  |
| Colon | 186 (65.7) | 13 (68.4) | 55 (54.5) | 118 (72.4) | 0.010 |
| Rectum | 97 (34.3) | 6 (6.5) | 46 (45.5) | 45 (27.6) |  |
| **T stage** |  |  |  |  |  |
| 1-3 | 183 (64.7) | 13 (68.4) | 62 (61.4) | 108 (66.3) | 0.679 |
| 4 | 100 (35.3) | 6 (31.6) | 39 (38.6) | 55 (33.7) |  |
| **N stage** |  |  |  |  |  |
| 0 | 120 (42.4) | 9 (47.4) | 36 (35.6) | 75 (46.0) | 0.231 |
| 1-2 | 163 (57.6) | 10 (52.6) | 65 (64.4) | 88 (54.0) |  |
| **Histological grade** |  |  |  |  |  |
| Well/moderate | 251 (88.7) | 17 (89.5) | 91 (90.1) | 143 (87.7) | 0.909 |
| Poor | 32 (11.3) | 2 (10.5) | 10 (9.9) | 20 (12.3) |  |
| **Liver metastases tumor size (cm)** |  |  |  |  |  |
| ≤3 | 197 (69.6) | 17 (89.5) | 80 (79.2) | 100 (61.3) | 0.001 |
| >3 | 86 (30.4) | 2 (10.5) | 21 (20.8) | 63 (38.7) |  |
| **Liver metastases number** |  |  |  |  |  |
| Single | 135 (47.7) | 12 (63.2) | 52 (51.5) | 71 (43.6) | 0.180 |
| Multiple | 148 (52.3) | 7 (36.8) | 49 (48.5) | 92 (56.4) |  |
| **Hepatic resection timing** |  |  |  |  |  |
| Metachronous | 119 (42.0) | 8 (42.1) | 46 (45.5) | 65 (39.9) | 0.693 |
| Synchronous | 164 (58.0) | 11 (57.9) | 55 (54.5) | 98 (60.1) |  |
| **Preoperative chemotherapy** |  |  |  |  |  |
| Yes | 143 (50.5) | 9 (47.4) | 50 (49.5) | 84 (51.5) | 0.910 |
| No | 140 (49.5) | 10 (52.6) | 51 (50.5) | 79 (48.5) |  |
| **Postoperative chemotherapy** |  |  |  |  |  |
| Yes | 218 (77.0) | 17 (89.5) | 80 (79.2) | 121 (74.2) | 0.291 |
| No | 65 (23.0) | 2 (10.5) | 21 (20.8) | 42 (25.8) |  |
| **CEA (ng/mL)** |  |  |  |  |  |
| ≤5 | 118 (41.7) | 11 (57.9) | 46 (45.5) | 61 (37.4) | 0.148 |
| >5 | 165 (58.3) | 8 (42.1) | 55 (54.5) | 102 (62.6) |  |
| **CA199 (U/mL)** |  |  |  |  |  |
| ≤37 | 213 (75.3) | 16 (84.2) | 78 (77.2) | 119 (73.0) | 0.485 |
| >37 | 69 (24.4) | 3 (15.8) | 22 (21.8) | 44 (27.0) |  |
| Not available | 1 (0.4) | 0 (0) | 1 (1) | 0 (0) |  |

Abbreviations: CEA, carcinoembryonic antigen; CA19-9, cancer antigen 19-9.
